# Health status and academic progress: A cross-sectional study of nursing students in Croatia and Serbia

**DOI:** 10.1590/1980-220X-REEUSP-2025-0082en

**Published:** 2025-09-22

**Authors:** Sunčica Ivanović, Ilija Kocić, Biljana Kocić, Milena Cvetković Jovanović, Vojin Vidanović, Snježana Čukljek, Boris Ilić, Janko Babić, Irena Kovačević

**Affiliations:** 1 College of Applied Health Sciences Ćuprija, Public Health, Ćuprija, Republic of Serbia.; 2 University Clinical Center Niš, Center for Mental Health Protection, Republic of Serbia.; 3 University of Niš, Faculty of Medicine Niš, Narrow Scientific Field Epidemiology, Republic of Serbia.; 4 University of Niš, Faculty of Medicine Niš, Employment at the Institute for Emergency Medicine in Niš, Republic of Serbia.; 5 College for Vocational Studies in Healthcare “Medica”, Belgrade, Republic of Serbia.; 6 University of Applied Health Sciences, Department of Nursing, Zagreb, Republic of Croatia.; 7 University of Rijeka, Faculty of Health Studies, Department of Nursing, Rijeka, Republic of Croatia.; 8 University of Applied Health Sciences, Department of Health Psychology, Zagreb, Republic of Croatia.

**Keywords:** Students, Nursing, Education, Nursing, Health Status, Academic progress, Estudantes de Enfermagem, Educação em Enfermagem, Nível de Saúde, Progresso acadêmico

## Abstract

**Objective:**

To compare the health status and academic progress of nursing students in Croatia, a European Union member state, and Serbia, a European Union candidate country, and to identify factors influencing their well-being and academic success.

**Method:**

A cross-sectional study was conducted with 424 nursing students from the University of Applied Health Sciences in Zagreb, Croatia, and the Academy of Educational and Medical Vocational Studies in Ćuprija, Serbia. Data were collected through an online questionnaire assessing health behavior, well-being, and academic performance. Statistical analyses included the Chi-square and Mann-Whitney U tests.

**Results:**

Croatian students had lower absenteeism (31.6%) and exhibited more responsible sexual health behaviors, while Serbian students reported higher levels of self-satisfaction (mean rank: 189.16 vs. 148.75; p < 0.01) and academic motivation (mean rank: 137.51 vs. 105.66; p < 0.01). Both groups showed health behaviors within normal ranges, though stress and substance use were prevalent. Statistically significant differences were found in self-satisfaction and academic progress (p < 0.05).

**Conclusion:**

This study highlights differences in health and academic outcomes between nursing students in Croatia and Serbia, influenced by cultural, educational, and familial factors. Interventions addressing stress and promoting healthy behaviors are recommended.

## RESUMO

**Objetivo:**

O objetivo deste trabalho é comparar o estado de saúde e o progresso acadêmico dos estudantes dos cursos de Ciências da Saúde na Croácia, Estado-membro da União Europeia, e na Sérvia, país candidato à adesão à União Europeia, bem como identificar os fatores que influenciam o seu bem-estar e sucesso acadêmico.

**Método:**

Foi realizado um estudo transversal com uma amostra de 424 estudantes dos cursos de Ciências da Saúde da Universidade de Ciências Aplicadas à Saúde (Croácia) e da Academia de Estudos Educacionais e Médicos Vocacionais em Ćuprija (Sérvia). Os dados foram coletados por meio de um questionário online que incluía a avaliação do comportamento de saúde, do bem-estar subjetivo e do desempenho acadêmico. Para a análise estatística, foram utilizados o teste do qui-quadrado e o teste de Mann-Whitney U.

**Resultados:**

Os estudantes croatas apresentaram uma menor taxa de faltas às aulas (31,6%) e demonstraram comportamentos mais responsáveis em relação à saúde sexual, enquanto os estudantes da Sérvia mostraram um nível mais alto de autoconfiança (média das classificações: 189,16 versus 148,75; p < 0,01) e de motivação acadêmica (média das classificações: 137,51 versus 105,66; p < 0,01). Ambos os grupos apresentaram padrões de comportamento de saúde dentro dos limites normais, embora o estresse e o uso de substâncias psicoativas tenham sido frequentes. Diferenças estatisticamente significativas foram encontradas no nível de autoconfiança e no progresso acadêmico (p < 0,05).

**Conclusão:**

Este estudo aponta para diferenças nos desfechos de saúde e acadêmicos entre os estudantes de cuidados de saúde na Croácia e na Sérvia, influenciadas por fatores culturais, educacionais e familiares. Recomenda-se a implementação de intervenções voltadas para a redução do estresse e a promoção de estilos de vida saudáveis.

## INTRODUCTION

The complexities of modern society, coupled with the increasing proportion of the elderly population and the prevalence of chronic non-communicable diseases, necessitate more sophisticated approaches to healthcare provision. It is crucial to enhance the overall healthcare of the population, as traditional healthcare systems are often inadequate to meet the evolving demands. In the context of healthcare delivery, there has been a growing emphasis on the advancement of nursing practice, resulting in the development of highly educated nursing professionals in numerous countries. Implementing innovations in healthcare services has required the integration of highly qualified personnel, leading to improved access to care, systematic evaluation and measurement of service quality, better health outcomes, and reduced healthcare costs^(1)^.

The significance of the role of nurses was particularly highlighted by the COVID-19 pandemic, which posed a global challenge and provided nurses with an opportunity for greater visibility, making them more recognized for their contributions to decision-making, addressing new challenges, and delivering high-quality, safe care to patients and their families^(2)^. The International Council of Nurses (ICN) warns of a shortage of highly educated nurses^(3)^. Despite the growth of this profession, the ICN still considers this increase inadequate to meet global demand^(1)^.

This global issue is affecting many countries, particularly within the European Union (EU) and the European Economic Area (EEA), which are grappling with a critical workforce shortage in healthcare. To address this, these countries are increasingly relying on the recruitment of qualified nurses from both within and outside the EU/EEA. The advantage for EU/EEA member states lies in the free movement of workers among these countries, facilitating the globalization of the nursing workforce. According to the European Parliament’s Directive on Professional Qualifications, there exists a system for the automatic recognition of nursing qualifications from these countries^(4,5)^. Furthermore, this increased mobility is also a result of active recruitment efforts in these countries to fill vacant positions. EU/EEA member states benefit from shared goals, cooperation, curriculum alignment, standardization, workforce planning, and easier employment opportunities^(3)^. Conversely, the situation in candidate countries seeking EU membership is markedly different, presenting additional challenges in engaging nurses both domestically and within EU/EEA member states.

Croatia is a member state of the European Union (EU) and, by European qualifications under EU Directive 2005/36/EC, enacted legislation in 2014 concerning undergraduate nursing studies. This initiative aimed not only to standardize study programs within the country but also to ensure a sufficient number of professionals who are not only qualified but also competent across all phases of the healthcare process. Through these legislative measures, Croatia has enabled future nursing students to practice not only in Croatia but also in all EU member states. Furthermore, Croatia has achieved alignment of educational outcomes as a signatory to the Bologna Declaration since 2001, which also led to the creation of the Dublin Descriptors^(6)^.

In Serbia, the signing of the Bologna Process was primarily a political decision, seen as a necessary reform due to the inefficacy of the previous system, which resulted in a significant number of students extending their studies or discontinuing their education. Serbia faces a serious challenge as nursing schools are proliferating, leading to a frequent occurrence of incompetent personnel, which raises concerns about the quality of education and the knowledge of newly graduated students^(7)^.

Despite the focus on education and workforce challenges, the health status of nursing students, a crucial component of their ability to perform academically and clinically, remains underexamined. Nursing students, much like their peers in other fields, are susceptible to various health risks, including poor dietary habits, physical inactivity, and mental health issues such as stress and anxiety^(8)^. Research indicates that students in healthcare programs, including nursing, often experience high levels of stress, which can negatively impact both their physical and mental well-being^(9,10,11,12)^. The demands of balancing academic workloads with clinical practice contribute to an increased prevalence of burnout among these students^(13,14)^.

Furthermore, academic progress among nursing students is influenced by various factors such as class attendance, study habits, and engagement in extracurricular activities. High levels of academic stress and challenges with time management are common among nursing students, which may lead to lower academic performance^(9)^. On the other hand, participation in clinical exercises and timely submission of assignments is positively associated with academic success^(15)^. These factors underline the importance of understanding how this population’s health and academic performance are interconnected.

For this study, we define highly educated nurses (ICN) as those who have attained the title of nurse, either general or specialist, through master’s education, acquiring a foundational body of professional knowledge, complex decision->making skills, and clinical competencies necessary for ­practicing nursing at an advanced level^(3)^. The evolving demands of healthcare systems, driven by an aging population and the prevalence of chronic diseases, highlight the critical role of highly educated nursing professionals^(1,2)^. Despite efforts to standardize nursing education within the European Union (EU) and align curricula in candidate countries, disparities persist in workforce mobility and educational quality^(4,5)^. This study addresses a gap in the literature by exploring the intersection of health behaviors and academic performance among nursing students^(9,13)^. These factors underline the importance of understanding how this population’s health and academic performance are interconnected.

**Therefore, this study** aims to compare the health status and academic progress of nursing students in Croatia, a European Union member state, and Serbia, a candidate country, to identify factors influencing their well-being and academic success.”

## METHOD

### Type and Place of Study

This cross-sectional study was conducted at two higher education institutions for nursing students: the University of Applied Health Sciences in Zagreb, Croatia, and the Academy of Educational and Medical Vocational Studies, Department of Medical Studies in Ćuprija, Serbia. These institutions were selected based on their similar curricular structure and their relevance as representative institutions within their respective national contexts.

### Participants

The study involved a total of 424 undergraduate nursing students. Participants were recruited using a convenience sampling method. All students enrolled in the first, second, or third year of undergraduate nursing studies at the participating institutions were invited to participate voluntarily. The choice to include only students from these three years reflects the structure of the Nursing programs in both countries, which are typically completed within three years, making this group representative of the full undergraduate nursing student population.

**The inclusion criterion was age between 18 and 48. There were no specific exclusion criteria.**

### Instruments

The instrument used in this study was the Student Health Questionnaire and additional questions related to academic progress, adapted from the study by Jacob and Kaushik^(9)^. The questionnaire was developed in collaboration with several healthcare institutions in the USA and was adapted for this study. The questions covered various aspects of students’ health and academic progress. The Student Health Questionnaire is an instrument designed to collect data related to students’ physical health, emotional state, safety, sexual activity, substance use, and academic progress. This questionnaire is used to assess students’ overall well-being with the goal of providing support and improving their health and academic success.

### Sections of the Questionnaire:

#### Sociodemographic Data

**This section contains basic information about the students, such as name, surname, date of birth, year of study, and social identity. It also includes data on students’ demographic profile, including questions about family, living conditions, and social support, which helps better understand the context in which students are placed. These data are useful for the analysis of other aspects of health and well-being.**

(Questions 1–3) – The questionnaire also includes data on students’ demographic profile, including questions about family, living conditions, and social support, which helps better understand the context in which students are placed. These data are useful for the analysis of other aspects of health and well-being.

***Perceptions of Student Health*** (Questions 4–13) – This section focuses on the student’s physical health and general well->being. It includes questions related to physical activity levels, nutrition, sleep patterns, dental health, doctor visits, and any current or past health issues (such as chronic conditions or acute symptoms).

***Safety and Injuries*** (Questions 14–18) – This section examines safety-related behaviors and experiences. It includes questions about wearing seatbelts, using helmets, texting or using a phone while driving, and any history of being threatened, injured, or experiencing violence at home or in school.

***Emotional State*** (Questions 19–23) – This section addresses the student’s emotional well-being. It includes questions about stress, anxiety, depressive symptoms, self-harm, and thoughts of suicide.

***Relationships and Sexual Activity*** (Questions 24–25) – This section covers sexual activity, including questions about the use of contraception and concerns regarding sexually transmitted infections.

***Habits and Substance Use*** (Questions 26–27) – This section explores the student’s substance use patterns, including alcohol, cigarette smoking, marijuana, and other drugs. It also addresses risky behaviors related to substance use.

***Academic Progress*** (Questions 1–15) – This section focuses on the student’s academic habits, class attendance, study behaviors, engagement with the learning process, and use of educational resources.

### Metric Characteristics

The questionnaire is based on validated scales and instruments, including PHK-2^(16)^ and CRAFFT^(17)^, which are recognized as effective tools for assessing student health and behavior. Each section has specific questions that allow for precise analysis of various dimensions of student health and well-being. As the questionnaires were not validated on the population on which this research was conducted, the metric characteristics of the test were examined.

Confirmatory Factor Analysis (CFA) was conducted to evaluate the factor structure of the adapted questionnaire. The scales exhibited an acceptable level of model fit, with χ^2^ values not being statistically significant. The Goodness-of-Fit Index (GFI) ranged from 0.95 to 0.96, the Adjusted Goodness-of-Fit Index (AGFI) from 0.90 to 0.93, the RMSEA from 0.05 to 0.07, and the CFI from 0.93 to 0.98. Cronbach’s alpha coefficients for the subscales ranged from 0.72 to 0.77, confirming internal consistency.

An Exploratory Factor Analysis (EFA, Principal Component Method) was also conducted. Promax Rotation was chosen as the choice of rotation, because all the factors represent an assessment of students’ health habits and academic success, so it is assumed that they correlate with each other. On that occasion, 6 factors were extracted using both the Gutman Kaiser criterion and the Scree Plot criterion, and those 6 factors explain 72.48% of the variance. In the Structure Matrix, the data shows that the items are organized by factors as well as the test key preorders.

### Scoring

The health status in this questionnaire is based on students’ self-reports. For scoring purposes, all questions that required recoding were recoded so that a higher score indicates a greater degree of the measured scales. Based on students’ responses, certain results may indicate potential risks to physical or mental health. For example, responses related to substance use and emotional state may suggest the need for additional counseling or support.

### Data Collection Process

The questionnaire was administered online, and the purpose of the study was explained to the students, ensuring their anonymity. The average time required to complete the questionnaire was approximately 10 minutes. Questionnaires were collected via electronic platforms without the possibility of identifying the respondents. **The data collection was conducted between January 8 and February 27, 2024.**

### Data Analysis

The statistical methods employed in the study included descriptive statistics, such as minimum and maximum values, arithmetic mean, and standard deviation, to summarize the data. The Chi-square test was used to analyze categorical variables, while the Mann-Whitney U test was applied to continuous variables. Confirmatory Factor Analysis (CFA) and Exploratory Factor Analysis (EFA) was conducted to evaluate the factor structure of the questionnaire, and Cronbach’s alpha test was used to assess the reliability of the scale. Additionally, the Kolmogorov-Smirnov test was performed to determine the normality of the data distribution. A significance level of 0.05 was established for all statistical tests.

### Ethical Aspects

The Ethics Committee of the University of Applied Health Sciences in Zagreb and the Academy of Educational and Medical Vocational Studies in Ćuprija both approved the study. All participants provided informed consent before participating in the study.

## RESULTS

A total of 424 respondents participated in the study, evenly distributed across the two faculties from which they originate: the University of Applied Health Sciences in Zagreb, Croatia (212; 50%) and the Academy of Educational and Medical Vocational Studies, Department of Medical Studies in Ćuprija, Serbia (Table 1). The majority of respondents were first-year students (184; 43.4%), followed by third-year students (140; 33%) and second-year students (97; 22.9%).

**Table 1:** Sample structure overview of nursing students (n = 424), from Zagreb (Croatia) and Ćuprija (Serbia), 2024.

Academic Institution		Frequency		Percent %	
Croatia			212		50
Serbia			212		50
Year of Study			Frequency	Percent %	Valid Percent %
Valid	1st year (undergraduate)		184	43.4	43.7
	2nd year (undergraduate)		97	22.9	23
	3rd year (undergraduate)		140	33	33.3
	Total		421	99.3	100
Missing	System		3	0.7	
Gender			Frequency	Percent %	Valid Percent %
Valid	Male		46	10.8	11
	Female		370	87.3	88.5
	Transgender Person		1	0.2	0.2
	Non-Binary person		1	0.2	0.2
	Total		418	98.6	100
Missing	System		6	1.4	
Living Situation			Frequency	Percent %	Valid Percent %
Valid	Parents and/or siblings		310	73.1	78.7
	Extended family		11	2.6	2.8
	Relatives		7	1.7	1.8
	Partner or partner and children		14	3.3	3.6
	Single parent with children and/or grandparents		23	5.4	5.8
	Roommate		14	3.3	3.6
	Living alone		15	3.6	3.5
	Total		394	92.9	100
Missing	System		30	7.1	
Total			424	100	
		Min	Max.	Mean	Std. Deviation
	Age Statistics	18	48	21.62	4.76

The sample comprised a higher number of females (370; 87.3%) and heterosexual individuals (365; 86.1%). The number of males was lower (46; 10.8%), but the sample also included one transgender individual (0.2%) and one non-binary individual (0.2%). Regarding sexual orientation, in addition to the predominant heterosexual respondents, the sample included homosexual individuals (18; 4.2%), bisexual individuals (17; 4%), pansexual individuals (1; 0.2%), as well as those who identified as uncertain (14; 3.3%).

A significant portion of respondents (310; 73.1%) lived in their primary families with both parents and/or with siblings. The least represented group consisted of those living with relatives (7; 1.7%). The age range of respondents spanned from 18 to 48 years, with an average age of 21.62 ± 4.76 years.

As shown in Table 2, this study examined the health and academic aspects of students from two educational institutions. Among the health aspects, the following were assessed: General Health Perception (M = 5.58, SD = 1.14), Sense of Safety from Potential Injury (M = 7.23, SD = 0.88), Sense of Well-Being (M = 3.51, SD = 1.04), Healthy Sexual Activity (M = 5.90, SD = 0.92), Use of Psychoactive Substances (M = 1.68, SD = 1.62), and Self- Satisfaction (M = 5.00, SD = 1.12). Regarding academic aspects, the tendency for academic progress was evaluated (M = 10.17, SD = 2.42).

**Table 2:** Overview of descriptive indicators of examined concepts, Zagreb (Croatia) and Ćuprija (Serbia), 2024.

	Minimum	Maximum	Mean	Std. Deviation	p Kolmogorov - Smirnov	Cronbach’s Alpha
Perception of general health	3	9	5.58	1.14	0.00	0.73
Sense of security from potential injury	4	9	7.23	0.88	0.00	0.75
Sense of Well-being	0	5	3.51	1.04	0.00	0.72
Healthy Sexual Activity	2	8	5.90	0.92	0.00	0.76
Use of Psychoactive Substances (PAS)	0	7	1.68	1.62	0.00	0.76
Self-Satisfaction	1	6	5	1.12	0.00	0.77
Academic Progress	4	15	10.17	2.42	0.04	0.74

The scales demonstrated low but satisfactory reliability (Cronbach’s α > 0.7). The statistical significance of the Kolmogorov-Smirnov test (p < 0.05) indicates that the responses on the scales do not follow a normal distribution. Consequently, the Mann-Whitney U test was employed for continuous variables, while the Chi-square test was used for categorical variables.

**Table 3** presents only the statistically significant differences. It was found that students from Croatia differ from those in Serbia regarding the presence of disputes within their family environment (χ^2^ = 12.24, df = 1, p < 0.01) and the existence of any issues in the family setting (χ^2^ = 7.43, df = 1, p = 0.01). Differences were also observed in terms of class attendance (χ^2^ = 5.32, df = 1, p < 0.05), conversations with friends (χ^2^ = 14.06, df = 1, p < 0.01), and discussions with parents (χ^2^ = 10.76, df = 1, p < 0.01).

**Table 3:** Overview of the relationship between sources of stress at home and college and social support concerning the Institutions Zagreb (Croatia) and Ćuprija (Serbia), 2024.

*X* ^2^ *=* 12.24; df = 1; p = 0.00			Institution	
			Croatia	Serbia
Family arguments	No	Count	144	177
		% of no arguments	44.9%	55.1%
	Yes	Count	58	30
		% of arguments	65.9%	34.1%
***X* ^2^ *=* 7.43; df = 1; p = 0.01**			**Institution**	
			**Croatia**	**Serbia**
No family problems	No	Count	76	52
		% no family problem	59.4%	40.6%
	Yes	Count	126	155
		% family problem	44.8%	55.2%
***X* ^2^ *=* 5.32; df = 1; p = 0.02**			**Institution**	
			**Croatia**	**Serbia**
Problems at university - Missed classes	No	Count	190	181
		% of no Problems at university – Missed classes	51.2%	48.8%
	Yes	Count	12	26
		% of Problems at university – Missed classes	31.6%	68.4%
***X* ^2^ *=* 14.06; df = 1; p = 0.00**			**Institution**	
			**Croatia**	**Serbia**
Frequency in communication with friends	No	Count	42	78
		% no Conversations with friends	35.0%	65.0%
	Yes	Count	160	129
		% of Conversations with friends	55.4%	44.6%
***X* ^2^ *=* 10.76; df = 1; p = 0.00**			**Institution**	
			**Croatia**	**Serbia**
Frequency in communication with parent	No	Count	88	58
		% no Conversations with Parent	60.3%	39.7%
	Yes	Count	114	149
		% Conversations with Parent	43.3%	56.7%

Legend: * X^2^ – Chi-square; df – degrees of freedom; p – statistical significance.

Croatian students reported a higher incidence of disputes in their family environment (58; 65.9%), while fewer indicated that they do not experience any problems at home (126; 44.8%). Additionally, they reported a lower rate of class absenteeism (12; 31.6%), more frequent conversations with friends (160; 55.4%), and less frequent discussions with their parents (114; 43.3%) compared to Serbian students.

**Table 4** presents only the variables for which statistically significant differences were identified. Students from Zagreb and those from Ćuprija exhibit statistically significant differences in their Sense of Well-Being (7945; p < 0.05), Healthy Sexual Behavior (1584; p = 0.01), Self-Satisfaction (10951; p < 0.01), and Academic Progress (5481; p < 0.01). Serbian students reported higher scores in Sense of Well-Being, greater self- satisfaction, and a stronger desire for academic advancement. In contrast, Croatian students demonstrated more responsible behavior concerning sexual activity. In all other cases, no statistically significant differences were found (p > 0.05).

**Table 4:** Overview of differences in the level of expression of certain aspects of health and academic progress concerning the Institution-Zagreb (Croatia), Ćuprija (Serbia), 2024.

	Institution	Mean Rank	Mann-Whitney U	p
	Croatia	123.64		
Sense of Well-being	Serbia	142.06	7495	0.04
	Croatia	72.70		
Healthy Sexual activity	Serbia	56.96	1584	0.01
	Croatia	148.75		
Self-satisfaction	Serbia	189.16	10951	0.00
	Croatia	105.66		
Academic progress	Serbia	137.51	5481	0.00

Legend: * p – statistical significance.

## DISCUSSION

The results of this study show that among students in higher education, in the study programs of Professional Medical Nurse and Nursing, there is a difference between health-related aspects (Sense of well-being, Healthy sexual activity, and Self-satisfaction) and academic aspects (Tendency for academic progress) in both higher education institutions. At the same time, students from both higher education institutions exhibit similar behaviors that fall within the normal, desirable, and expected range when considering risks associated with lifestyle. In nursing education establishments, the regular curriculum includes specific subjects and methodological units aimed at educating young individuals about the importance of adopting healthy lifestyles.

The determination of health indicators is the responsibility of socioeconomic factors, lifestyle choices, and the physical environment. The World Health Organization (WHO) identifies several risk factors associated with lifestyle choices, including unhealthy diet, lack of physical activity, sleep disturbances, exposure to stressful situations, smoking, alcohol consumption, and caffeine intake^(1)^. It is not without reason that Confucius stated that one should prepare the support they will rely on in old age during their youth. Nursing education emphasizes the early prevention of all health risk factors. In this context, risky behaviors during youth can influence later lifestyle-related health risks.

One of the pressing global issues impacting the youth population is the widespread use of psychoactive and illicit substances among students, with a significant number of young individuals having either experimented with or contemplated trying such substances^(18)^. Behaviors related to lifestyle risks were generally within acceptable ranges for students at both institutions. While 81% of students reported alcohol use in the past 12 months, substance use overall remained within acceptable ranges, consistent with previous studies^(13,18)^.

Nevertheless, it is essential to consider that respondents’ answers regarding health behaviors among young people may not always reflect maximum honesty, particularly concerning the use of psychoactive substances (PAS). In daily interactions and work with young people and the student population, inadequate and problematic behaviors, as well as unsuitable lifestyles, can often be observed, emphasizing the continuous need for health promotion among the student demographic^(9)^. When it comes to healthy eating, the majority of youth today tend to opt for fast food, pastries, or skip meals, and exhibit lower levels of physical activity. The categorization of psychoactive substances, tobacco, and alcohol complicates assessment due to the diversity of substances, terms, frequencies of use, and categories employed for grouping substances^(18)^.

### Well-being, Self-satisfaction, and Academic Progress

The results of our study revealed statistically significant differences between students from Serbia and Croatia in terms of their sense of well-being, healthy sexual behavior, self-satisfaction, and academic progress. Serbian students exhibited higher scores in their sense of well-being, reported greater self-satisfaction, and expressed a stronger desire for academic advancement. These findings align with previous research by Jacob and Kaushik^(9)^, which highlights the importance of motivational and emotional factors in academic success among nursing students^(9)^. Recent studies have demonstrated a connection between academic progress, general health, and health-related quality of life (HRQoL) among students^(19,20)^. Academic performance positively influences life satisfaction and fosters a sense of happiness among students, which, in turn, affects their self-perception of quality of life^(19)^. Furthermore, research by Qi et al. supports the link between general health perceptions and academic performance, particularly in high-pressure academic environments such as healthcare education^(19)^.

### Croatian Students – Academic Responsibility and Emotional Burden

In contrast, Croatian students demonstrated greater academic responsibility and more conscientious behavior regarding sexual relationships. However, they also reported higher levels of family conflict and lower scores in self-satisfaction and well-being. This may reflect the greater emotional and academic burden placed on highly engaged students in their studies and clinical responsibilities. Previous literature suggests that excessive academic stress can negatively affect mental health and overall life satisfaction^(8,15)^. The relatively lower self-reported well-being among Croatian students could also be linked to the dual burden of institutional demands and family expectations.

### Family Communication, Risk Behaviors, and Sexual Health

Our findings indicate that Croatian students report more frequent conflicts with their parents but are more engaged in conversations with friends. While family conflict may be associated with emotional distress, peer support has been identified as a protective factor in mitigating risky behaviors, particularly related to sexual activity^(21)^. Croatian students’ more responsible sexual behavior could be influenced by greater institutional awareness campaigns or by peer norms. Meanwhile, Serbian students, many of whom live far from home, might face fewer direct parental interactions, which potentially reduces their stress but also limits emotional support. Studies show that family communication is associated with safer sexual practices and greater emotional resilience among youth.

The differences observed between Croatian and Serbian nursing students can also be contextualized through the structural characteristics of their respective educational systems, as outlined in the Introduction. Croatia, as a member of the European Union, has implemented standardized nursing curricula following the European Directive 2005/36/EC, ensuring alignment with EU educational and professional qualification standards. This harmonization may contribute to the higher academic responsibility observed among Croatian students, as the system places strong emphasis on competence and cross->border employability within the EU.

In contrast, Serbia’s nursing education system, while formally aligned with the Bologna Process, continues to face challenges related to educational quality and institutional consistency. The proliferation of nursing schools without adequate regulatory oversight may create disparities in the academic preparedness and motivation of students. These contextual differences likely influence not only the students’ academic progress but also their well-being, expectations, and coping mechanisms throughout their studies.

By reintroducing these systemic distinctions in the Discussion, we can offer a deeper understanding of how educational policy and institutional frameworks shape nursing students’ experiences and outcomes in both countries.

## STUDY LIMITATIONS

The findings of this study cannot be generalized to all nursing students, as the sample includes only one institution in each of the two studied countries. This limited sample may not fully represent the diversity of nursing students across different educational settings, cultural contexts, or geographic areas.

Additionally, the use of an online questionnaire introduces several limitations. The self-selection nature of voluntary participation could lead to selection bias, as students with particular interests or motivations may have been more likely to respond. Furthermore, the online format, which relied on mobile phone distribution, may have introduced response biases due to variations in access or ease of use. This method of distribution also limits control over data collection, potentially affecting the representativeness of the sample.

There is also a risk of social desirability bias in responses to sensitive questions, especially those related to sexuality, substance use, and experiences of violence or abuse. These topics may carry cultural taboos, particularly in the Republic of Serbia, where public discussions on such subjects are relatively recent. Consequently, students may have hesitated to disclose personal information fully, leading to potential underreporting or inaccuracies in responses.

Additional mediating or confounding variables, such as socioeconomic background, mental health status, and academic pressure, were not controlled for in this study. These factors could independently influence both health status and academic progress, thereby affecting the reliability of the findings.

Finally, the cross-sectional design of the study limits the ability to infer causality between the identified factors and observed outcomes. Longitudinal studies would provide more robust insights into how health behaviors and academic progress evolve and the lasting impact of these behaviors on students’ well-being and performance.

## CONCLUSION

This study provides a comparative analysis of the health status and academic progress of nursing students in Croatia and Serbia. The findings indicate notable differences between the two groups: Serbian students reported higher levels of self-satisfaction and academic motivation, while Croatian students demonstrated lower absenteeism and more responsible sexual health behaviors. These outcomes appear to reflect broader cultural, educational, and institutional contexts in both countries.

While our results suggest certain patterns, such as the potential interplay between well-being and academic engagement, it is important to interpret these findings with caution. The data were based on self- reported perceptions, which may be subject to response biases. Moreover, the cross-sectional design of the study limits causal inference.

Thus, although associations between health aspects and academic progress are evident, causal interpretations should be approached with caution. Instead, we highlight the need for further longitudinal and mixed-methods research to explore these dynamics more comprehensively.

Tailored institutional interventions that promote student well-being and academic support, especially in relation to stress management, family support, and health education, are recommended to address the identified challenges and enhance student outcomes.

## Data Availability

The dataset used in this study is available from the authors upon reasonable request. The data are not publicly available as they involve collaboration between multiple researchers from different institutions, including partners from Croatia, and in order to protect the personal data of the participants. All data access requests will be reviewed in accordance with the ethical principles and research guidelines of the project.
