# Supplementary material for: Health status and academic progress: A cross-sectional study of nursing students in Croatia and Serbia
Source: Rev Esc Enferm USP. 2025 Sep 22;59:e20250082. doi: 10.1590/1980-220X-REEUSP-2025-0082en (PMC12453549; doi:10.1590/1980-220X-REEUSP-2025-0082en)
Supplement: Translation

# Translation: Estado de salud y progreso académico: un estudio transversal de estudiantes de enfermería en Croacia y Serbia

**DOI:** 10.1590/1980-220X-REEUSP-2025-0082es

**Authors:** Sunčica Ivanović, Ilija Kocić, Biljana Kocić, Milena Cvetković Jovanović, Vojin Vidanović, Snježana Čukljek, Boris Ilić, Janko Babić, Irena Kovačević

**Affiliations:** 1 College of Applied Health Sciences Ćuprija, Public Health, Ćuprija, Republic of Serbia.; 2 University Clinical Center Niš, Center for Mental Health Protection, Republic of Serbia.; 3 University of Niš, Faculty of Medicine Niš, Narrow Scientific Field Epidemiology, Republic of Serbia.; 4 University of Niš, Faculty of Medicine Niš, Employment at the Institute for Emergency Medicine in Niš, Republic of Serbia.; 5 College for Vocational Studies in Healthcare “Medica”, Belgrade, Republic of Serbia.; 6 University of Applied Health Sciences, Department of Nursing, Zagreb, Republic of Croatia.; 7 University of Rijeka, Faculty of Health Studies, Department of Nursing, Rijeka, Republic of Croatia.; 8 University of Applied Health Sciences, Department of Health Psychology, Zagreb, Republic of Croatia.

**Keywords:** Estudiantes de Enfermería, Educación en Enfermería, Estado de Salud, Progreso académico

## Abstract

**Objetivo:**

Comparar el estado de salud y el progreso académico de los estudiantes de enfermería en Croacia, un Estado miembro de la Unión Europea, y en Serbia, un país candidato a la adhesión a la Unión Europea, e identificar los factores que influyen en su bienestar y en su éxito académico.

**Método:**

Se realizó un estudio transversal con 424 estudiantes de enfermería de la Universidad de Ciencias de la Salud Aplicadas de Zagreb (Croacia) y de la Academia de Estudios Educativos y Médicos Profesionales de Ćuprija (Serbia). Los datos se recolectaron mediante un cuestionario en línea que evaluaba el comportamiento en materia de salud, el bienestar y el rendimiento académico. Los análisis estadísticos incluyeron las pruebas de chi cuadrado y U de Mann-Whitney.

**Resultados:**

Los estudiantes croatas tuvieron un menor absentismo (31,6%) y mostraron comportamientos más responsables en materia de salud sexual, mientras que los estudiantes serbios informaron de mayores niveles de autosatisfacción (rango medio: 189,16 frente a 148,75; p < 0,01) y motivación académica (rango medio: 137,51 frente a 105,66; p < 0,01). Ambos grupos mostraron comportamientos de salud dentro de los rangos normales, aunque prevalecían el estrés y el consumo de sustancias. Se encontraron diferencias estadísticamente significativas en la autosatisfacción y el progreso académico (p < 0,05).

**Conclusión:**

Este estudio destaca las diferencias en los resultados académicos y de salud entre los estudiantes de enfermería de Croacia y Serbia, influidas por factores culturales, educativos y familiares. Se recomiendan intervenciones que aborden el estrés y promuevan comportamientos saludables.

## INTRODUCCIÓN

La complejidad de la sociedad moderna, junto con el aumento de la proporción de personas mayores y la prevalencia de enfermedades crónicas no transmisibles, exige enfoques más sofisticados para la prestación de asistencia sanitaria. Es fundamental mejorar la asistencia sanitaria general de la población, ya que los sistemas sanitarios tradicionales suelen ser insuficientes para satisfacer las demandas cambiantes. En el contexto de la prestación de servicios de salud, se ha hecho cada vez más hincapié en el avance de la práctica de la enfermería, lo que ha dado lugar a la formación de profesionales de enfermería altamente cualificados en numerosos países. La aplicación de innovaciones en los servicios de salud ha requerido la integración de personal altamente cualificado, lo que ha mejorado el acceso a la atención, la evaluación y la medición sistemáticas de la calidad de los servicios, los resultados en materia de salud y la reducción de los costos de la atención de salud^(1)^.

La importancia del papel de las enfermeras se puso especialmente de relieve con la pandemia de COVID-19, que supuso un reto mundial y brindó a las enfermeras la oportunidad de ganar visibilidad, lo que les permitió obtener un mayor reconocimiento por su contribución a la toma de decisiones, la respuesta a nuevos retos y la prestación de una atención de alta calidad y segura a los pacientes y sus familias^(2)^. El Consejo Internacional de Enfermeras (CIE) advierte de la escasez de enfermeras con un alto nivel de formación^(3)^. A pesar del crecimiento de esta profesión, el CIE sigue considerando que este aumento es insuficiente para satisfacer la demanda mundial^(1)^.

Este problema mundial afecta a muchos países, en particular a los de la Unión Europea (UE) y del Espacio Económico Europeo (EEE), que se enfrentan a una grave escasez de personal sanitario. Para hacer frente a esta situación, estos países recurren cada vez más a la contratación de enfermeras cualificadas tanto dentro como fuera de la UE/EEE. La ventaja para los Estados miembros de la UE/EEE radica en la libre circulación de trabajadores entre estos países, lo que facilita la globalización de la mano de obra de enfermería. De acuerdo con la Directiva del Parlamento Europeo sobre cualificaciones profesionales, existe un sistema de reconocimiento automático de las cualificaciones de enfermería de estos países^(4,5)^. Además, esta mayor movilidad es también el resultado de los esfuerzos activos de contratación en estos países para cubrir los puestos vacantes. Los Estados miembros de la UE/EEE se benefician de objetivos comunes, cooperación, armonización de los planes de estudios, normalización, planificación de la mano de obra y mayores oportunidades de empleo^(3)^. Por el contrario, la situación de los países candidatos a la adhesión a la UE es muy diferente, ya que se enfrentan a retos adicionales para contratar enfermeros tanto a nivel nacional como en los Estados miembros de la UE/EEE.

Croacia es un Estado miembro de la Unión Europea (UE) y, en virtud de las cualificaciones europeas establecidas en la Directiva 2005/36/CE de la UE, promulgó en 2014 una legislación relativa a los estudios de enfermería de grado. Esta iniciativa tenía por objeto no solo normalizar los programas de estudios en el país, sino también garantizar un número suficiente de profesionales que no solo estuvieran cualificados, sino también competentes en todas las fases del proceso sanitario. Gracias a estas medidas legislativas, Croacia ha permitido a los futuros estudiantes de enfermería ejercer no solo en Croacia, sino también en todos los Estados miembros de la UE. Además, Croacia ha logrado la armonización de los resultados educativos como signataria de la Declaración de Bolonia desde 2001, lo que también condujo a la creación de los Descriptores de Dublín^(6)^.

En Serbia, la firma del Proceso de Bolonia fue principalmente una decisión política, considerada una reforma necesaria debido a la ineficacia del sistema anterior, que provocó que un número significativo de estudiantes prolongaran sus estudios o abandonaran su formación. Serbia se enfrenta a un grave reto, ya que las escuelas de enfermería están proliferando, lo que da lugar a una frecuente aparición de personal incompetente, lo que suscita preocupación por la calidad de la educación y los conocimientos de los recién graduados^(7)^.

A pesar de la atención prestada a los retos en materia de educación y mano de obra, el estado de salud de los estudiantes de enfermería, un componente crucial de su capacidad para rendir académica y clínicamente, sigue sin examinarse en profundidad. Los estudiantes de enfermería, al igual que sus compañeros de otros campos, son susceptibles a diversos riesgos para la salud, entre ellos los malos hábitos alimenticios, la inactividad física y los problemas de salud mental, como el estrés y la ansiedad^(8)^. Las investigaciones indican que los estudiantes de programas de atención sanitaria, incluida la enfermería, suelen experimentar altos niveles de estrés, lo que puede afectar negativamente a su bienestar físico y mental^(9-12)^. Las exigencias de conciliar la carga académica con la práctica clínica contribuyen a una mayor prevalencia del agotamiento entre estos estudiantes^(13,14)^.

Además, el progreso académico de los estudiantes de enfermería se ve influido por diversos factores, como la asistencia a clase, los hábitos de estudio y la participación en actividades extracurriculares. Los altos niveles de estrés académico y los retos que plantea la gestión del tiempo son comunes entre los estudiantes de enfermería, lo que puede dar lugar a un rendimiento académico inferior^(9)^. Por otra parte, la participación en ejercicios clínicos y la entrega puntual de los trabajos se asocian positivamente con el éxito académico^(15)^. Estos factores subrayan la importancia de comprender cómo están interrelacionados la salud y el rendimiento académico de esta población.

Para este estudio, definimos a las enfermeras con un alto nivel de formación (CIE) como aquellas que han obtenido el título de enfermera, ya sea general o especializada, a través de una formación de máster, adquiriendo un conjunto de conocimientos profesionales básicos, habilidades de toma de decisiones complejas y competencias clínicas necesarias para ejercer la enfermería a un nivel avanzado^(3)^. Las exigencias cambiantes de los sistemas de salud, impulsadas por el envejecimiento de la población y la prevalencia de enfermedades crónicas, ponen de relieve el papel fundamental de los profesionales de enfermería con un alto nivel de formación^(1,2)^. A pesar de los esfuerzos por normalizar la formación en enfermería en la Unión Europea (UE) y armonizar los planes de estudios en los países candidatos, persisten las disparidades en la movilidad de la mano de obra y la calidad de la educación^(4,5)^. Este estudio aborda una laguna en la literatura al explorar la intersección entre los comportamientos de salud y el rendimiento académico de los estudiantes de enfermería^(9,13)^. Estos factores subrayan la importancia de comprender cómo se interrelacionan la salud y el rendimiento académico de esta población.

Por lo tanto, este estudio tiene como objetivo comparar el estado de salud y el progreso académico de los estudiantes de enfermería en Croacia, un Estado miembro de la Unión Europea, y Serbia, un país candidato, para identificar los factores que influyen en su bienestar y éxito académico.

## MÉTODO

### Tipo y Lugar del Estudio

Este estudio transversal se llevó a cabo en dos instituciones de educación superior para estudiantes de enfermería: la Universidad de Ciencias de la Salud Aplicadas de Zagreb (Croacia) y la Academia de Estudios Educativos y Médicos Profesionales, Departamento de Estudios Médicos de Ćuprija (Serbia). Estas instituciones fueron seleccionadas por su estructura curricular similar y su relevancia como instituciones representativas en sus respectivos contextos nacionales.

### Participantes

En el estudio participaron un total de 424 estudiantes de grado de enfermería. Los participantes fueron seleccionados mediante un método de muestreo por conveniencia. Se invitó a participar de forma voluntaria a todos los estudiantes matriculados en el primer, segundo o tercer año de los estudios de grado de enfermería en las instituciones participantes. La decisión de incluir solo a estudiantes de estos tres años refleja la estructura de los programas de enfermería en ambos países, que suelen completarse en tres años, lo que hace que este grupo sea representativo de la población total de estudiantes de grado de enfermería.

El criterio de inclusión fue tener entre 18 y 48 años. No hubo criterios de exclusión específicos.

### Instrumentos

El instrumento utilizado en este estudio fue el Cuestionario de Salud Estudiantil y preguntas adicionales relacionadas con el progreso académico, adaptado del estudio de Jacob y Kaushik^(9)^. El cuestionario fue desarrollado en colaboración con varias instituciones de salud de los Estados Unidos y se adaptó para este estudio. Las preguntas abarcaban diversos aspectos de la salud y el progreso académico de los estudiantes. El Cuestionario de Salud del Estudiante es un instrumento diseñado para recolectar datos relacionados con la salud física, el estado emocional, la seguridad, la actividad sexual, el consumo de sustancias y el progreso académico de los estudiantes. Este cuestionario se utiliza para evaluar el bienestar general de los estudiantes con el objetivo de brindarles apoyo y mejorar su salud y su éxito académico.

### Secciones del Cuestionario:

#### Datos Sociodemográficos

Esta sección contiene información básica sobre los estudiantes, como el nombre, los apellidos, la fecha de nacimiento, el año de estudio y la identidad social. También incluye datos sobre el perfil demográfico de los estudiantes, con preguntas sobre la familia, las condiciones de vida y el apoyo social, lo que ayuda a comprender mejor el contexto en el que se encuentran los estudiantes. Estos datos son útiles para el análisis de otros aspectos de la salud y el bienestar.

(Preguntas 1–3) – El cuestionario también incluye datos sobre el perfil demográfico de los estudiantes, incluyendo preguntas sobre la familia, las condiciones de vida y el apoyo social, lo que ayuda a comprender mejor el contexto en el que se encuentran los estudiantes. Estos datos son útiles para el análisis de otros aspectos de la salud y el bienestar.

***Percepciones de la salud de los estudiantes*** (Preguntas 4–13): esta sección se centra en la salud física y el bienestar general de los estudiantes. Incluye preguntas relacionadas con los niveles de actividad física, la nutrición, los patrones de sueño, la salud dental, las visitas al médico y cualquier problema de salud actual o pasado (como enfermedades crónicas o síntomas agudos).

***Seguridad y lesiones*** (Preguntas 14–18): esta sección examina los comportamientos y experiencias relacionados con la seguridad. Incluye preguntas sobre el uso del cinturón de seguridad, el uso del casco, el envío de mensajes de texto o el uso del teléfono mientras se conduce, y cualquier antecedente de amenazas, lesiones o violencia en el hogar o en la escuela.

***Estado emocional*** (preguntas 19–23): esta sección aborda el bienestar emocional del estudiante. Incluye preguntas sobre el estrés, la ansiedad, los síntomas depresivos, las autolesiones y los pensamientos suicidas.

***Relaciones y actividad sexual*** (Preguntas 24–25): esta sección abarca la actividad sexual, incluyendo preguntas sobre el uso de anticonceptivos y preocupaciones relacionadas con las infecciones de transmisión sexual.

***Hábitos y consumo de sustancias*** (Preguntas 26–27): esta sección explora los patrones de consumo de sustancias del estudiante, incluyendo el alcohol, el tabaco, la marihuana y otras drogas. También aborda los comportamientos de riesgo relacionados con el consumo de sustancias.

***Progreso académico*** (preguntas 1–15): esta sección se centra en los hábitos académicos del estudiante, la asistencia a clase, los comportamientos de estudio, la participación en el proceso de aprendizaje y el uso de los recursos educativos.

### Características de Medición

El cuestionario se basa en escalas e instrumentos validados, entre los que se incluyen PHK-2^(16)^ y CRAFFT^(17)^, reconocidos como herramientas eficaces para evaluar la salud y el comportamiento de los estudiantes.

Cada sección contiene preguntas específicas que permiten un análisis preciso de diversas dimensiones de la salud y el bienestar de los estudiantes. Dado que los cuestionarios no se validaron en la población en la que se llevó a cabo esta investigación, se examinaron las características métricas de la prueba.

Se realizó un análisis factorial confirmatorio (AFC) para evaluar la estructura factorial del cuestionario adaptado. Las escalas mostraron un nivel aceptable de ajuste del modelo, con valores χ^2^ no estadísticamente significativos.

El índice de bondad de ajuste (Goodness-of-Fit Index - GFI) osciló entre 0,95 y 0,96, el índice de bondad de ajuste ajustado (AGFI) entre 0,90 y 0,93, el RMSEA entre 0,05 y 0,07 y el CFI entre 0,93 y 0,98. Los coeficientes alfa de Cronbach para las subescalas oscilaron entre 0,72 y 0,77, lo que confirma la consistencia interna.

También se llevó a cabo un análisis factorial exploratorio (EFA, método de componentes principales). Se eligió la rotación Promax como opción de rotación, ya que todos los factores representan una evaluación de los hábitos de salud y el rendimiento académico de los estudiantes, por lo que se supone que están correlacionados entre sí. En esa ocasión, se extrajeron 6 factores utilizando tanto el criterio de Gutman Kaiser como el criterio de Scree Plot, y esos 6 factores explican el 72,48% de la varianza. En la matriz de estructura, los datos muestran que los ítems están organizados por factores, así como por el orden de las claves de la prueba.

### Puntuación

El estado de salud en este cuestionario se basa en los autoinformes de los estudiantes. A efectos de puntuación, todas las preguntas que requerían recodificación se recodificaron de manera que una puntuación más alta indicara un mayor grado en las escalas medidas. Según las respuestas de los estudiantes, algunos resultados pueden indicar riesgos potenciales para la salud física o mental. Por ejemplo, las respuestas relacionadas con el consumo de sustancias y el estado emocional pueden sugerir la necesidad de asesoramiento o apoyo adicional.

### Proceso de Recolección de Datos

El cuestionario se administró en línea y se explicó a los estudiantes el propósito del estudio, garantizando su anonimato. El tiempo medio necesario para completar el cuestionario fue de aproximadamente 10 minutos. Los cuestionarios se recolectaron a través de plataformas electrónicas sin posibilidad de identificar a los encuestados. La recolección de datos se llevó a cabo entre el 8 de enero y el 27 de febrero de 2024.

### Análisis de Datos

Los métodos estadísticos empleados en el estudio incluyeron estadísticas descriptivas, tales como valores mínimos y máximos, media aritmética y desviación estándar, para resumir los datos. Se utilizó la prueba de chi cuadrado para analizar las variables categóricas, mientras que se aplicó la prueba U de Mann-Whitney a las variables continuas. Se llevó a cabo un análisis factorial confirmatorio (AFC) y un análisis factorial exploratorio (AFE) para evaluar la estructura factorial del cuestionario, y se utilizó la prueba alfa de Cronbach para evaluar la fiabilidad de la escala. Además, se realizó la prueba de Kolmogorov-Smirnov para determinar la normalidad de la distribución de los datos. Se estableció un nivel de significación de 0,05 para todas las pruebas estadísticas.

### Aspectos Éticos

El Comité de Ética de la Universidad de Ciencias de la Salud Aplicadas de Zagreb y la Academia de Estudios Educativos y Médicos Profesionales de Ćuprija aprobaron el estudio. Todos los participantes dieron su consentimiento informado antes de participar en el estudio.

## RESULTADOS

En el estudio participaron un total de 424 encuestados, distribuidos de manera uniforme entre las dos facultades de las que proceden: la Universidad de Ciencias de la Salud Aplicadas de Zagreb, Croacia (212; 50%) y la Academia de Estudios Educativos y Médicos Profesionales, Departamento de Estudios Médicos de Ćuprija, Serbia (Tabla 1). La mayoría de los encuestados eran estudiantes de primer año (184; 43,4%), seguidos de los de tercer año (140; 33%) y los de segundo año (97; 22,9%).

**Tabla 1:** Estructura de la muestra de estudiantes de enfermería (n = 424), de Zagreb (Croacia) y C´uprija (Serbia), 2024.

Institución académica		Frecuencia		Porcentaje	
Croacia			212		50
Serbia			212		50
Año de estudio			Frecuencia	Porcentaje	Porcentaje válido %
Válido	1.º año (grado)		184	43,4	43
	2.º año (grado)		97	22,9	23
	3.º año (grado)		140	33	33
	Total		421	99,3	100
Faltan	Sistema		3	0,7	
Género			Frecuencia	Porcentaje %	Porcentaje válido %
Válido	Masculino		46	10,8	11
	Mujer		370	87,3	88,5
	Persona transgénero		1	0,2	0,2
	Persona no binaria		1	0,2	0,2
	Total		418	98,6	100
Faltan	Sistema		6	1,4	
Situación de convivencia			Frecuencia	Porcentaje %	Porcentaje válido %
Válido	Padres y/o hermanos		310	73,1	78,7
	Familia extensa		11	2,6	2,8
	Parientes		7	1,7	1,8
	Pareja o pareja e hijos		14	3,3	3,6
	Familias monoparentales con hijos y/o abuelos		23	5,4	5,8
	Compañero de piso		14	3,3	3,6
	Vive solo		15	3,6	3,5
	Total		394	92,9	100
Faltan	Sistema		30	7,1	
Total			424	100	
		Mín	Máx	Media	Desviación Desviación
	Estadísticas de edad	18	48	21,62	4,76

La muestra estaba compuesta por un mayor número de mujeres (370; 87,3%) y personas heterosexuales (365; 86,1%). El número de hombres era menor (46; 10,8%), pero la muestra también incluía una persona transgénero (0,2%) y una persona no binaria (0,2%). En cuanto a la orientación sexual, además de los encuestados heterosexuales, que eran la mayoría, la muestra incluía personas homosexuales (18; 4,2%), bisexuales (17; 4%), pansexuales (1; 0,2%), así como personas que se identificaban como indecisas (14; 3,3%).

Una parte significativa de los encuestados (310; 73,1%) vivía en su familia principal con ambos padres y/o con hermanos. El grupo menos representado era el de los que vivían con familiares (7; 1,7%). La edad de los encuestados oscilaba entre los 18 y los 48 años, con una media de 21,62 ± 4,76 años.

Como se muestra en la Tabla 2, este estudio examinó los aspectos académicos y de salud de los estudiantes de dos instituciones educativas. Entre los aspectos de salud, se evaluaron los siguientes: percepción general de la salud (M = 5,58, DE = 1,14), sensación de seguridad frente a posibles lesiones (M = 7,23, DE = 0,88), sensación de bienestar (M = 3,51, DE = 1,04), actividad sexual saludable (M = 5,90, DE = 0,92), consumo de sustancias psicoactivas (M = 1,68, DE = 1,62) y autosatisfacción (M = 5,00, DE = 1,12). En cuanto a los aspectos académicos, se evaluó la tendencia al progreso académico (M = 10,17, DE = 2,42).

**Tabla 2:** Resumen de los indicadores descriptivos de los conceptos examinados, Zagreb (Croacia) y Ćuprija (Serbia), 2024.

	Mínimo	Máximo	Media	Desvío estándar (DE)	p Kolmogorov - Smirnov	Alfa de Cronbach
Percepción de la salud general	3	9	5,58	1,14	0	0,73
Sensación de seguridad frente a posibles lesiones	4	9	7,23	0,88	0	0,75
Sensación de bienestar	0	5	3,51	1,04	0	0,72
Salud sexual Actividad	2	8	5,90	0,92	0,00	0,76
Uso de sustancias psicoactivas	0	7	1,68	1,62	0	0,76
Autosatisfacción	1	6	5	1,12	0	0,77
Progreso académico	4	15	10,17	2,42	0,04	0,74

Las escalas demostraron una fiabilidad baja pero satisfactoria (α de Cronbach > 0,7). La significación estadística de la prueba de Kolmogorov-Smirnov (p< o 0,05) indica que las respuestas en las escalas no siguen una distribución normal. En consecuencia, se empleó la prueba U de Mann-Whitney para las variables continuas, mientras que se utilizó la prueba de chi cuadrado para las variables categóricas.

**La Tabla 3** presenta solo las diferencias estadísticamente significativas. Se observó que los estudiantes de Croacia difieren de los de Serbia en cuanto a la presencia de disputas en su entorno familiar (χ^2^ = 12,24, df = 1, p < 0,01) y la existencia de problemas en el entorno familiar (χ^2^ = 7,43, df = 1, p = 0,01). También se observaron diferencias en cuanto a la asistencia a clase (χ^2^ = 5,32, df = 1, p < 0,05), las conversaciones con amigos (χ^2^ = 14,06, df = 1, p < 0,01) y las conversaciones con los padres (χ^2^ = 10,76, df = 1, p < 0,01).

**Tabla 3:** Resumen de la relación entre las fuentes de estrés en el hogar y la universidad y el apoyo social en relación con las instituciones Zagreb (Croacia) y Ćuprija (Serbia), 2024.

*X* ^2^ *=* 12,24; df = 1; p = 0,00			Institución	
			Croacia	Serbia
Discusiones familiares	No	Recuento	144	177
		% sin discusiones	44,9	55,1
	Sí	Recuento	58	30
		% de argumentos	65,9	34,1
***X* ^2^ *=* 7,43; df = 1; p = 0,01**			**Institución**	
			**Croacia**	**Serbia**
Sin problemas familiares	No	Recuento	76	52
		% sin problemas familiares	59,4	40,6
	Sí	Recuento	126	155
		% de problemas familiares	44	55,2
***X* ^2^ *=* 5,32; df = 1; p = 0,02**			**Institución**	
			**Croacia**	**Serbia**
Problemas en la universidad - Faltas a clase	No	Recuento	190	181
		% de no Problemas en la universidad – Faltas a clase	51,2	48,8
	Sí	Recuento	12	26
		% de problemas en la universidad – Ausencias clases	31,6	68,4
***X* ^2^ *=* 14,06; df = 1; p = 0,00**			**Institución**	
			**Croacia**	**Serbia**
Frecuencia en la comunicación con amigos	No	Recuento	42	78
		% no Conversaciones con amigos	35,0	65,0
	Sí	Contar	160	129
		% de conversaciones con amigos	55,4	44,6
***X* ^2^ *=* 10,76; df = 1; p = 0,00**			**Institución**	
			**Croacia**	**Serbia**
Frecuencia de comunicación con los padres	No	Recuento	88	58
		% sin conversaciones con los padres	60,3	39,7
	Sí	Recuento	114	149
		% Conversaciones con los padres	43,3	56

Leyenda: * X^2^ – Chi-cuadrado; df – grados de libertad; p – significación estadística.

Los estudiantes croatas informaron una mayor incidencia de conflictos en su entorno familiar (58; 65,9%), mientras que menos indicaron que no experimentaban ningún problema en casa (126; 44,8%). Además, informaron de un menor índice de absentismo escolar (12; 31,6%), conversaciones más frecuentes con amigos (160; 55,4%) y discusiones menos frecuentes con sus padres (114; 43,3%) en comparación con los estudiantes serbios.

**La Tabla 4** presenta solo las variables para las que se identificaron diferencias estadísticamente significativas. Los estudiantes de Zagreb y los de Ćuprija muestran diferencias estadísticamente significativas en su sensación de bienestar (7945; p < 0,05), comportamiento sexual saludable (1584; p = 0,01) y satisfacción personal (10951; p < 0,01) y progreso académico (5481; p < 0,01). Los estudiantes serbios obtuvieron puntuaciones más altas en sentido de bienestar, mayor autosatisfacción y un deseo más fuerte de progreso académico. Por el contrario, los estudiantes croatas mostraron un comportamiento más responsable en relación con la actividad sexual. En todos los demás casos, no se encontraron diferencias estadísticamente significativas (p < 0,05).

**Tabla 4:** Resumen de las diferencias en el nivel de expresión de determinados aspectos de la salud y el rendimiento académico en relación con la institución: Zagreb (Croacia), Ćuprija (Serbia), 2024.

	Institución	Rango medio	U de Mann-Whitney	p
	Croacia	123,64		
Sensación de bienestar	Serbia	142,06	7495	0,04
	Croacia	72,70		
Actividad sexual saludable	Serbia	56,96	1584	0,01
	Croacia	148,75		
Autosatisfacción	Serbia	189,16	10951	0,00
	Croacia	105,66		
Progreso académico	Serbia	137,51	5481	0,00

Leyenda: * p – significación estadística.

## DISCUSIÓN

Los resultados de este estudio muestran que entre los estudiantes de educación superior, en los programas de estudios de Enfermería Profesional Médica y Enfermería, existe una diferencia entre los aspectos relacionados con la salud (sensación de bienestar, actividad sexual saludable y satisfacción personal) y los aspectos académicos (tendencia al progreso académico) en ambas instituciones de educación superior. Al mismo tiempo, los estudiantes de ambas instituciones de educación superior muestran comportamientos similares que se encuentran dentro del rango normal, deseable y esperado cuando se consideran los riesgos asociados al estilo de vida. En los centros de formación en enfermería, el plan de estudios habitual incluye asignaturas específicas y unidades metodológicas destinadas a educar a los jóvenes sobre la importancia de adoptar estilos de vida saludables.

La determinación de los indicadores de salud es responsabilidad de factores socioeconómicos, elecciones de estilo de vida y el entorno físico. La Organización Mundial de la Salud (OMS) identifica varios factores de riesgo asociados con las elecciones de estilo de vida, entre ellos la alimentación poco saludable, la falta de actividad física, los trastornos del sueño, la exposición a situaciones estresantes, el tabaquismo, el consumo de alcohol y la ingesta de cafeína^(1)^. No en vano Confucio afirmó que hay que preparar en la juventud el apoyo en el que se apoyará en la vejez. La formación en enfermería hace hincapié en la prevención temprana de todos los factores de riesgo para la salud. En este contexto, los comportamientos de riesgo durante la juventud pueden influir en los riesgos para la salud relacionados con el estilo de vida en el futuro.

Uno de los problemas mundiales más acuciantes que afectan a la población joven es el consumo generalizado de sustancias psicoactivas e ilícitas entre los estudiantes, ya que un número significativo de jóvenes ha probado o ha pensado en probar estas sustancias^(18)^. Los comportamientos relacionados con los riesgos del estilo de vida se encontraban, en general, dentro de los límites aceptables para los estudiantes de ambas instituciones. Si bien el 81% de los estudiantes declararon haber consumido alcohol en los últimos 12 meses, el consumo de sustancias en general se mantuvo dentro de los límites aceptables, en consonancia con estudios anteriores^(13,18)^.

No obstante, es fundamental tener en cuenta que las respuestas de los encuestados sobre los comportamientos de salud de los jóvenes pueden no reflejar siempre la máxima honestidad, especialmente en lo que se refiere al consumo de sustancias psicoactivas. En las interacciones diarias y el trabajo con los jóvenes y la población estudiantil, a menudo se observan conductas inadecuadas y problemáticas, así como estilos de vida inadecuados, lo que pone de relieve la necesidad continua de promover la salud entre la población estudiantil^(9)^. En lo que respecta a la alimentación saludable, la mayoría de los jóvenes de hoy en día tienden a optar por la comida rápida, los pasteles o a saltarse comidas, y muestran niveles más bajos de actividad física. La categorización de las sustancias psicoactivas, el tabaco y el alcohol complica la evaluación debido a la diversidad de sustancias, términos, frecuencias de consumo y categorías empleadas para agruparlas^(18)^.

### Bienestar, Autosatisfacción y Progreso Académico

Los resultados de nuestro estudio revelaron diferencias estadísticamente significativas entre los estudiantes de Serbia y Croacia en términos de su sensación de bienestar, comportamiento sexual saludable, autosatisfacción y progreso académico. Los estudiantes serbios obtuvieron puntuaciones más altas en su sensación de bienestar, declararon una mayor autosatisfacción y expresaron un deseo más fuerte de progreso académico. Estos hallazgos coinciden con investigaciones anteriores de Jacob y Kaushik^(9)^, que destacan la importancia de los factores motivacionales y emocionales en el éxito académico de los estudiantes de enfermería^(9)^. Estudios recientes han demostrado una conexión entre el progreso académico, la salud general y la calidad de vida relacionada con la salud (CVRS) entre los estudiantes^(19,20)^. El rendimiento académico influye positivamente en la satisfacción con la vida y fomenta la sensación de felicidad entre los estudiantes, lo que, a su vez, afecta a su percepción de la calidad de vida^(19)^. Además, la investigación de Qi et al. respalda la relación entre la percepción general de la salud y el rendimiento académico, especialmente en entornos académicos de alta presión, como la formación sanitaria^(19)^.

### Estudiantes Croatas: Responsabilidad Académica y Carga Emocional

Por el contrario, los estudiantes croatas demostraron una mayor responsabilidad académica y un comportamiento más concienzudo en lo que respecta a las relaciones sexuales. Sin embargo, también informaron de mayores niveles de conflicto familiar y puntuaciones más bajas en satisfacción personal y bienestar. Esto puede reflejar la mayor carga emocional y académica que soportan los estudiantes muy comprometidos con sus estudios y sus responsabilidades clínicas. La bibliografía anterior sugiere que el estrés académico excesivo puede afectar negativamente a la salud mental y a la satisfacción general con la vida^(8,15)^. El bienestar relativamente más bajo declarado por los estudiantes croatas también podría estar relacionado con la doble carga de las exigencias institucionales y las expectativas familiares.

### Comunicación Familiar, Conductas de Riesgo y Salud Sexual

Nuestros hallazgos indican que los estudiantes croatas informan de conflictos más frecuentes con sus padres, pero están más comprometidos en las conversaciones con sus amigos. Si bien los conflictos familiares pueden estar asociados con el malestar emocional, el apoyo de los compañeros se ha identificado como un factor protector para mitigar las conductas de riesgo, en particular las relacionadas con la actividad sexual^(21)^. El comportamiento sexual más responsable de los estudiantes croatas podría estar influenciado por una mayor concienciación institucional o por las normas de los compañeros. Por su parte, los estudiantes serbios, muchos de los cuales viven lejos de casa, podrían tener menos interacción directa con sus padres, lo que podría reducir su estrés, pero también limitar el apoyo emocional. Los estudios demuestran que la comunicación familiar está asociada con prácticas sexuales más seguras y una mayor resiliencia emocional entre los jóvenes.

Las diferencias observadas entre los estudiantes de enfermería croatas y serbios también pueden contextualizarse a través de las características estructurales de sus respectivos sistemas educativos, como se describe en la introducción. Croacia, como miembro de la Unión Europea, ha implementado planes de estudios de enfermería estandarizados siguiendo la Directiva Europea 2005/36/CE, lo que garantiza la alineación con las normas educativas y de cualificación profesional de la UE. Esta armonización puede contribuir a la mayor responsabilidad académica observada entre los estudiantes croatas, ya que el sistema hace hincapié en la competencia y la empleabilidad transfronteriza dentro de la UE.

En contraste, el sistema de formación en enfermería de Serbia, aunque formalmente alineado con el Proceso de Bolonia, sigue enfrentándose a retos relacionados con la calidad educativa y la coherencia institucional. La proliferación de escuelas de enfermería sin una supervisión reguladora adecuada puede crear disparidades en la preparación académica y la motivación de los estudiantes. Es probable que estas diferencias contextuales influyan no solo en el progreso académico de los estudiantes, sino también en su bienestar, sus expectativas y sus mecanismos de afrontamiento a lo largo de sus estudios.

Al reintroducir estas diferencias sistémicas en el debate, podemos ofrecer una comprensión más profunda de cómo la política educativa y los marcos institucionales configuran las experiencias y los resultados de los estudiantes de enfermería en ambos países.

## LIMITACIONES DEL ESTUDIO

Los resultados de este estudio no pueden generalizarse a todos los estudiantes de enfermería, ya que la muestra incluye solo una institución en cada uno de los dos países estudiados. Esta muestra limitada puede no representar plenamente la diversidad de los estudiantes de enfermería en diferentes entornos educativos, contextos culturales o zonas geográficas.

Además, el uso de un cuestionario en línea introduce varias limitaciones. La naturaleza autoseleccionada de la participación voluntaria podría dar lugar a un sesgo de selección, ya que los estudiantes con intereses o motivaciones particulares podrían haber sido más propensos a responder. Además, el formato en línea, que se basó en la distribución de teléfonos móviles, puede haber introducido sesgos en las respuestas debido a las variaciones en el acceso o la facilidad de uso. Este método de distribución también limita el control sobre la recolección de datos, lo que podría afectar la representatividad de la muestra.

También existe el riesgo de sesgo de deseabilidad social en las respuestas a preguntas delicadas, especialmente las relacionadas con la sexualidad, el consumo de sustancias y las experiencias de violencia o abuso. Estos temas pueden conllevar tabúes culturales, especialmente en la República de Serbia, donde los debates públicos sobre estos temas son relativamente recientes. En consecuencia, es posible que los estudiantes hayan dudado en revelar toda su información personal, lo que podría dar lugar a un infrarregistro o a inexactitudes en las respuestas.

En este estudio no se controlaron otras variables mediadoras o de confusión, como el contexto socioeconómico, el estado de salud mental y la presión académica. Estos factores podrían influir de forma independiente tanto en el estado de salud como en el progreso académico, lo que afectaría a la fiabilidad de los resultados.

Por último, el diseño transversal del estudio limita la capacidad de inferir causalidad entre los factores identificados y los resultados observados. Los estudios longitudinales proporcionarían una visión más sólida de cómo evolucionan los comportamientos de salud y el progreso académico, así como del impacto duradero de estos comportamientos en el bienestar y el rendimiento de los estudiantes.

## CONCLUSIÓN

Este estudio ofrece un análisis comparativo del estado de salud y el progreso académico de los estudiantes de enfermería en Croacia y Serbia. Los resultados indican diferencias notables entre los dos grupos: los estudiantes serbios declararon niveles más altos de autosatisfacción y motivación académica, mientras que los croatas mostraron un menor absentismo y comportamientos más responsables en materia de salud sexual. Estos resultados parecen reflejar contextos culturales, educativos e institucionales más amplios en ambos países.

Si bien nuestros resultados sugieren ciertos patrones, como la posible interacción entre el bienestar y el compromiso académico, es importante interpretar estos hallazgos con cautela. Los datos se basaron en percepciones autoinformadas, que pueden estar sujetas a sesgos de respuesta. Además, el diseño transversal del estudio limita la inferencia causal.

Por lo tanto, aunque las asociaciones entre los aspectos de la salud y el progreso académico son evidentes, las interpretaciones causales deben abordarse con cautela. En su lugar, destacamos la necesidad de realizar más investigaciones longitudinales y con métodos mixtos para explorar estas dinámicas de forma más exhaustiva.

Se recomiendan intervenciones institucionales adaptadas que promuevan el bienestar de los estudiantes y el apoyo académico, especialmente en relación con la gestión del estrés, el apoyo familiar y la educación sanitaria, para abordar los retos identificados y mejorar los resultados de los estudiantes.

## Data Availability

El conjunto de datos utilizado en este estudio está disponible por parte de los autores previa solicitud razonable. Los datos no están disponibles públicamente ya que implican la colaboración de varios investigadores de diferentes instituciones, incluidos socios de Croacia, y con el fin de proteger los datos personales de los participantes. Todas las solicitudes de acceso a los datos serán evaluadas de acuerdo con los principios éticos y las directrices de investigación del proyecto.
